# Complete genome of *Enterobacter roggenkampii* VT5-1 harboring the IncFIA-type plasmid encoding *mcr-10* isolated from Vietnamese rice noodle

**DOI:** 10.1128/mra.00576-25

**Published:** 2026-01-30

**Authors:** Machika Saito, Yuki Kasumi, Tatsuya Nakayama

**Affiliations:** 1 Graduate School of Integrated Sciences for Life, Hiroshima University 12803, Higashi-Hiroshima, Hiroshima, Japan; Rochester Institute of Technology, Rochester, New York, USA

**Keywords:** colistin-resistant *Enterobacter roggenkampii*, *mcr-10*, plasmid, rice noodle, Vietnam

## Abstract

*Enterobacter roggenkampii* VT5-1 was isolated from rice noodles, an imported food product from Vietnam. The chromosome and plasmid from the strain were sequenced using Oxford Nanopore sequencing. Total genomes are approximately 4.9 Mbp long, and the IncFIA plasmid encoding *mcr-10* was detected in the strain.

## ANNOUNCEMENT

Plasmid-mediated antimicrobial resistance genes are frequently disseminated through horizontal gene transfer, which raises considerable concern due to their potential for rapid and widespread distribution. Here, we report the whole genome sequence (WGS) of *Enterobacter roggenkampii* harboring a plasmid encoding *mcr-10* isolated from rice noodle, an imported product from Vietnam.

**Fig 1:**
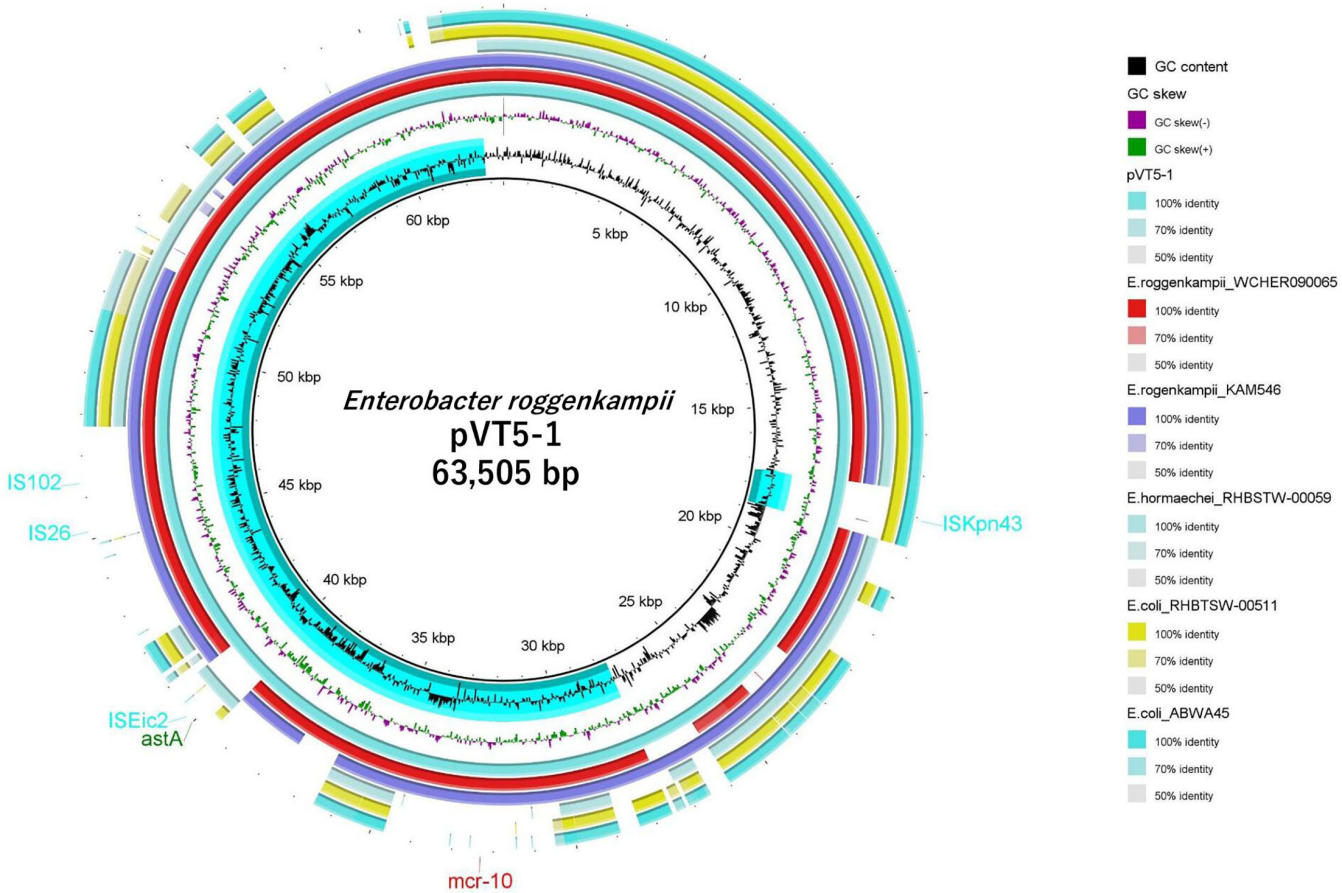
Genome map of *Enterobacter roggenkampii* VT5-1. Additional genomes were included for sequence identity comparison. Whole genome analysis revealed that *E. roggenkampii* VT5-1 carries *mcr-10* on a plasmid. The complete genomes of *E. roggenkampii* WCHER090065 (accession number CP045065.1), *E. roggenkampii* KAM546 (accession number AP026874.1), *E. hormaechei* RHBSTW-00059 (accession number CP058168.1), *E. coli* RHBSTW-00511 (accession number CP055919.1), and *E. coli* ABWA45 (accession number CP106963.1) were selected for the plasmid map of pVT5-1. Red markers indicate antimicrobial resistance genes; light blue markers represent insertion sequences; and green markers denote virulence genes.

Vietnamese rice noodles were purchased online in Higashi-Hiroshima, Hiroshima Prefecture, Japan, in 2024. A total of 25 g of rice noodle was mixed with 225 mL of buffered peptone water (composition [g/L]: peptone 10.0, sodium chloride 5.0, potassium dihydrogen phosphate 1.5, and di-sodium hydrogen phosphate dodecahydrate 9.0) (Merck, Darmstadt, Germany). After incubation at 37°C for 24 h, 100 µL of the culture was spread on MacConkey agar (Shimadzu Diagnostics Corporation, Tokyo, Japan) containing 2 µg/mL of colistin and incubated at 37°C for 24 h. Several red colonies were selected and initially identified as *Enterobacter* spp. by 16S rRNA gene sequencing (1). After subculturing in LB medium at 37°C for 18 h, DNA was extracted using the Nucleospin Microbial DNA (Takara Bio, Shiga, Japan). The DNA concentration was 67 ng/µL as measured by Quantus Fluorometer (Promega, Madison, USA). For sequencing library preparation, Rapid barcoding kit v.24 (Oxford Nanopore Technologies, Oxford, UK) was used, and sequencing was performed using MinION (Oxford) with flow cell R10.4.1 (Oxford). Long-read quality checks were performed with fastqc v.0.11.9 (2) and Nanofilt v.2.8.0 (3). Dorado v.0.8.3 was used as the base caller. MinION (total reads 366,326; *N*_50_ value 4,820,631; total bases 746.7 Mb) sequencing data were assembled using hybracter v.0.11.0 (4). Hybracter automatically reorients the chromosome to start at the dnaA locus during assembly using default parameters unless otherwise specified. Annotation was performed using DFAST v.1.2.0 software. The assembled WGS was initially analyzed using SpeciesFinder 2.0. However, due to its limited species-level accuracy, ANIb analysis was additionally performed to improve species identification reliability. Combined results from both approaches confirmed that the isolate belonged to *E. roggenkampii*. Furthermore, ResFinder v.4.7.2 identified the *mcr-10* gene, which showed high similarity to that of *E. roggenkampii* VT5-1, the strain characterized in this study (Fig. 1). Genome completeness was assessed using BUSCO v.6 with the bacteria_odb12 data set. The assembly contained 97.4% complete BUSCOs (including 96.6% single copy and 0.9% duplicated), with 0.9% fragmented and 1.7% missing genes, indicating high assembly completeness. Moreover, plasmid characterization using PlasmidFinder identified a single circular IncFIA-type plasmid. Sequence comparison revealed 98.7% identity with previously reported *Salmonella* Typhi R27 plasmids.

**TABLE 1:** Complete genome information on *Enterobacter roggenkampii* VT5-1

Parameter	Value for *E. roggenkampii* VT5-1	
No. of CDSs	4,638	
No. of rRNAs	25	
No. of tRNAs	84	
No. of contigs	1	
Sequence coverage	152.9×	
	**Chromosome**	**Plasmid pVT5**
Total length (bp)	4,820,631	63,505
GC content (%)	55.9	53.1
MLST/Inc type	ST1635	IncFIA
Antibiotic resistant gene(s)	*bla*_MIR_ *, fosA*	*mcr-10*
Accession number	AP044038	AP044039

Isolate of *E. roggenkampii* contributes to the food-borne spread of resistant bacteria across countries. Furthermore, the *mcr* gene family is known as mobile colistin resistance genes and has 10 known variants (*mcr-1 *to *mcr-10*). Since *mcr* genes are often located on plasmids, there is a possibility that these resistance genes may spread through horizontal gene transfer. The identification of an *mcr-10* carrying plasmid is significant because *mcr-10* is poorly characterized, and its plasmid-mediated mobility suggests a high potential for the unnoticed spread of colistin resistance. This finding highlights the epidemiological relevance of *mcr-10* harboring plasmids in Enterobacterales.

## Data Availability

The *Enterobacter roggenkampii* VT5-1 WGS genome sequence was deposited in DDBJ/GenBank (accession number AP044038). The raw reads were deposited under accession number DRX708331 with the BioSample number SAMD00900824 and BioProject number PRJDB18898 (Table 1).
